# Factors associated with the recurrence of intermittent exotropia and reoperations in the long term

**DOI:** 10.1080/07853890.2026.2620331

**Published:** 2026-03-31

**Authors:** Pedro Lino, Pedro Aguiar, João Paulo Cunha

**Affiliations:** a NOVA National School of Public Health, Public Health Research Centre, Comprehensive Health Research Center, CHRC, LA-REAL,CCAL, NOVA University Lisbon, Lisbon, Portugal; b Orthoptist. CUF Cascais Hospital and CUF Tejo Hospital -Portugal, Santa Maria Local Health Unit, Lisbon, Portugal; c Scientific Area of Orthoptics and Vision Sciences. Higher School of Technology and Health of Lisbon, Lisbon, Polytechnic Institute of Lisbon Portugal, Lisbon, Portugal; d Ophthalmologist. Coordinator of the Ophthalmology Unit at CUF Cascais Hospital, Cascais, Portugal; e Department of Morpho-Functional Sciences of the Higher School of Technology and Health of Lisbon, Polytechnic Institute of Lisbon, Lisbon, Portugal

**Keywords:** Intermittent exotropia, surgical success, recurrence, reoperation, paediatric strabismus

## Abstract

**Purpose:**

To evaluate long-term reoperation risk after bilateral lateral rectus (BLR) recession for intermittent exotropia (IXT) in a paediatric cohort, and to identify the perioperative factors associated with recurrence and reoperation.

**Methods:**

A retrospective observational cohort of 258 children with basic or divergence-excess IXT who underwent BLR recession at CUF Cascais Hospital between 2010 and 2020 was analysed. Clinical variables included age, age at surgery, pre- and immediate postoperative deviation angles, preoperative occlusion therapy, orthoptic treatment, binocular function, and initial surgical success (residual deviation <10 prism dioptres with fusion). Bivariate analyses were performed using t-tests or χ^2^ tests, and variables with *p* < 0.10 were entered into multivariable binary logistic regression to identify the independent predictors of reoperation. Model diagnostics included variance inflation factors (VIF), Hosmer–Lemeshow test, and area under the receiver operating characteristic curve (AUC). Significance was set at *p* < 0.05.

**Results:**

Reoperation was required in 11% of cases. Failed initial surgery (adjusted OR = 34.7; *p* < 0.001), larger preoperative deviation (adjusted OR per PD = 1.143; 95% CI 1.062–1.231; *p* < 0.001), larger immediate postoperative deviation (adjusted OR per PD = 0.822; 95% CI 0.766–0.881; *p* < 0.001), and older patient age (adjusted OR per year = 1.261; 95% CI 1.032–1.542; *p* = 0.024) were independently associated with reoperation. Orthoptic and occlusion therapies showed non-significant trends.

**Conclusion:**

In children undergoing BLR for IXT, initial surgical failure and deviation magnitude (pre- and immediate postoperative) are the strongest predictors of reoperation, with age having the modest additional effect. These findings emphasize precise surgical planning and the need for long-term follow-up.

## Introduction

Intermittent exotropia (IXT) is one of the most common types of childhood strabismus, with a reported prevalence of approximately 0.5–1.0% in paediatric populations and representing the majority of exotropias in children. It is characterized by an intermittent outward deviation of one or both eyes, typically manifesting at distance fixation and becoming more apparent during fatigue, inattention, or illness.

The pathophysiology of IXT is generally attributed to an imbalance between divergence drive and fusional convergence mechanisms. Children often maintain ocular alignment through adequate fusional reserves; however, these reserves can be insufficient, especially at distance, limiting the ability to maintain fusion under visual stress. Variations in the accommodative convergence to accommodation (AC/A) ratio and proximal convergence mechanisms may further contribute to decompensation over time, with episodes becoming more frequent or sustained as children grow, become fatigued, or face increased visual demands.

Clinically, loss of control in IXT can lead to sensory deficits such as suppression, reduced stereopsis, and impaired binocular vision. Visible misalignment may also impact psychosocial well-being, affecting self-esteem and social interactions. Although surgical correction – most commonly bilateral lateral rectus (BLR) recession – is generally effective in realigning the eyes, recurrence and the need for reoperation remain important concerns, particularly over long-term follow-up. Large registry-based studies report 5-year cumulative reoperation rates of around 21%, with other series indicating recurrence rates up to 44% depending on follow-up duration, surgical technique, and patient characteristics [1–4].

Several perioperative factors have been identified as potential predictors of recurrence. Larger preoperative deviation and immediate postoperative Undercorrection are associated with higher recurrence risk, whereas slight overcorrection or minimal residual deviation postoperatively is generally linked to improved long-term stability [5–9]. The choice of surgical technique may also influence outcomes: some studies suggest unilateral recession–resection (RR) may have lower reoperation rates compared with BLR, although other reports find no significant difference when initial deviation and patient age are controlled [10–12]. Age at surgery, preoperative binocular function, and the use of orthoptic or occlusion therapy have also been investigated, but evidence remains inconclusive [13–15].

Despite extensive literature, many previous studies are limited by heterogeneous inclusion criteria, varying surgical techniques, or relatively short follow-up periods. Few studies have systematically analysed perioperative clinical, sensory, and motor factors associated with reoperation at long-term follow-up [16,17].

This study addresses these gaps by examining a large, homogeneous cohort of 258 children with basic-type intermittent exotropia who underwent BLR recession at a single tertiary institution, with a minimum follow-up of five years. The primary objectives are to determine the cumulative risk of reoperation over five years and to identify clinical, sensory and surgical factors associated with the need for reoperation, providing a clearer understanding of long-term surgical outcomes in IXT.

## Methods

### Study design and population

This was a retrospective observational cohort study including 258 children who underwent BLR recession for basic or divergence-excess intermittent exotropia between 2010 and 2020 at CUF Cascais Hospital (Lisbon, Portugal). Baseline was defined as the date of the first BLR procedure. Patients were followed for up to 5 years postoperatively.

All operations were performed at the Ophthalmology Unit, CUF Cascais Hospital. Surgeries were carried out by same surgical team (principal surgeon, adjutant surgeon and anaesthesiologist) -experienced surgeons with expertise in paediatric strabismus. All surgeons adhered to a standardized institutional protocol for BLR recession - Surgical dosage for BLR recession was determined according to the Parks nomogram.

Only primary bilateral lateral rectus (BLR) recessions were included in this cohort – w use only this technique because is more favourable for binocular vision and not produce lateral in concomitances. Patients undergoing unilateral recess-resect procedures, medial rectus resections, or other horizontal muscle surgeries were excluded. Reoperations included any subsequent horizontal muscle surgery (including RR or MR resections) performed for residual or recurrent exotropia.

Orthoptic therapy was offered postoperatively to patients with suboptimal fusion or residual divergence. The standard regimen at our centre comprised: Office-based sessions (30–45 min) every 1–2 weeks for 6–12 weeks focusing on fusional convergence (synoptophore and vectogram exercises) and accommodative facility training and Home-based exercises (pencil push-ups and convergence brock string or home vectogram practice) prescribed daily for 10–15 min, with recommended frequency of 5–7 days/week.

Because the compliance in therapy, in statistical analyst only included patients who are doing therapy program with convenience (good adhesion in the office and home therapy).

### Data collection

Clinical data were obtained from electronic medical records. Evaluations included clinical history, visual acuity, ocular motility, and binocular sensory testing using a synoptophore. These tests were executed by same orthoptist and with guidelines of PEDIG GROUP and American Academy of Pediatric Ophthalmology and strabismus. After surgery, some patients underwent orthoptic therapy aimed at improving fusion and convergence. Preoperative alternate occlusion was used as an anti-suppression exercise. In patients with alternating deviation and no marked fixation preference, alternate occlusion was applied for 1 h per day, whereas in patients with unilateral deviation and strong fixation preference, occlusion of the dominant eye was performed for 1 h per day. Postoperative orthoptic therapy included in-office exercises performed at the treatment room and using a synoptophore, aimed at strengthening fusion and convergence, complemented by home-based exercises to reinforce these skills.

### Variables analysed

The study analysed perioperative variables potentially associated with reoperation, including: preoperative occlusion therapy, preoperative deviation angle (prism diopters, PD), postoperative deviation angle (PD) – immediate postoperative deviation was defined as the ocular deviation measured at the first postoperative visit, performed within 24–72 h after surgery, prior to initiation of orthoptic therapy., binocular function (present/absent), surgical success (initial surgical success was defined as ocular alignment within <10 prism diopters of eso- or exodeviation with demonstrable fusion, assessed at the first postoperative visit (within 1 week after surgery). Surgical failure was defined as a residual deviation ≥10 prism diopters and/or absence of fusion at this same time point), orthoptic treatment (yes/no), patient age at surgery, and sex.

Reoperation was defined as any secondary horizontal muscle surgery performed for residual or recurrent exotropia.

### Statistical analysis

All analyses were performed using IBM SPSS Statistics version 26 (IBM Corp., Armonk, NY, USA). Continuous variables are presented as mean ± standard deviation (SD), and categorical variables as counts and percentages.

For description on sample, we did a univariate analysis. Second, bivariate comparisons were conducted using Student’s t-test or Mann–Whitney U test for continuous variables, and χ^2^ or Fisher’s exact test for categorical variables. Variables with *p* < 0.10 in bivariate analyses and those considered clinically relevant were entered into multivariable binary logistic regression models to identify independent predictors of reoperation (outcome: reoperation yes/no).

Backward stepwise elimination (entry *p* = 0.05, removal *p* = 0.10) was used to build the final model. Multicollinearity was assessed with variance inflation factors (VIF > 5 indicating collinearity). Model fit was evaluated using the Hosmer–Lemeshow goodness-of-fit test, and discrimination was assessed with the area under the receiver operating characteristic (ROC) curve (AUC).

Two-sided *p*-values < 0.05 were considered statistically significant.

### Ethical consideration

The study protocol was approved by the Ethics Comité of the CUF Health Group (approval number 2024/808) and conducted in accordance with the Declaration of Helsinki. Given it retrospective design, the Ethics Committee waived the requirement for additional informed consent. However, written informed consent for data use in research and education was obtained from parents or legal guardians at the time of surgery. Assent from children was generally not obtained due to their young age.

All data were collected retrospectively from existing clinical records and pseudo-anonymized prior to analysis to ensure participant privacy. No identifiable personal data were used, and all procedures adhered strictly to institutional and national guidelines for confidentiality and data protection. Every step of the study respected the ethical standards required for clinical research.

## Results

### Cohort characteristics

A total of 258 children who underwent bilateral lateral rectus (BLR) recession for intermittent exotropia were included. The mean age at baseline surgery was 8.7 ± 4.8 years (min 8 yo and max 18 yo). Overall, 11% (*n* = 29) of patients required reoperation during the five-year follow-up period. Among female patients, 13.3% required reoperation, compared with 7.6% of male patients; however, this difference was not statistically significant (*p* = 0.154). Baseline demographic and clinical characteristics by reoperation status are presented in Table 2.

### Preoperative and postoperative alignment

Before surgery, the mean angle of exodeviation was significantly larger in patients who later required reoperation compared to those who did not (38.5 ± 7.6 prism diopters [PD] vs. 30.9 ± 7.0 PD; *p* < 0.001). At the first postoperative assessment, residual deviation was also significantly greater among patients who subsequently underwent reoperation (12.3 ± 7.7 PD vs. 2.5 ± 5.8 PD; *p* < 0.001).

Surgical failure, defined as residual deviation ≥10 PD or loss of fusion, was strongly associated with reoperation. Seventy per cent of patients with initial surgical failure required a second procedure, compared with only 6.3% of those with successful alignment (*p* < 0.001).

### Preoperative sensory and therapeutic factors

Preoperative occlusion therapy was performed in 13.0% of patients who later required reoperation and in 2.3% of those who did not. Orthoptic therapy was conducted postoperatively in 9.4% of reoperated patients and 12.4% of non-reoperated patients, showing no statistically significant difference (*p* = 0.456). Absence of preoperative binocular function was observed in 33.3% of reoperated cases compared to 10.7% of non-reoperated cases; this difference did not reach significance (*p* = 0.109).

### Univariate analysis

In univariate logistic regression analyses, several variables showed significant associations with reoperation (Table 1).

**Table 1.** Parks nomogram for exotropia surgery.

Deviation (PD)	BLRc	LRc + MRs (R&R)
15	4.0	4.0 + 3.0
20	5.0	5.0 + 4.0
25	6.0	6.0 + 4.0
30	7.0	7.0 + 5.5
35	7.5	7.5 + 5.5
40	8.0	8.0 + 5.5
45	8.5	8.5 + 6.0
50	9.0	9.0 + 6.5
60	9.0 + 7.0	—

**Table 2.** Baseline characteristics and univariate analysis of factors associated with reoperation (*n* = 258).

Variable	No Reoperation (*n* = 229)	Reoperation (*n* = 29)	OR (95% CI)	*p*-value
**Sex**				
** Female Sex (ref)**	144 (86,7%)	22 (13,3%)	0.536 (0.228–1.260)	0.154
** **Male Sex	85(92,4%)	7(7,6%)		
** Age at surgery (years)**	8.3 ± 4.6	9.6 ± 5.2	1.066 (0.998–1.138)	0.055†
** Preoperative deviation (PD)**	30.9 ± 7.0	38.5 ± 7.6	1.151 (1.068–1.241)	<0.001***
** Immediate postoperative deviation (PD)**	2.5 ± 5.8	12.3 ± 7.7	0.797 (0.735–0.865)	<0.001***
**Surgical success**				
** Yes**	223 (93,%)	14 (70%)	0.029 (0.010–0.086)	<0.001***
** **No	6 (30%)	15 (6,3%)		
**Preoperative occlusion therapy**				
** No (Ref.)**	42 (97,7%)	1 (23,3)	6.289 (0.801–49.359)	0.075†
** Yes**	187 (87%)	28 (13,3%)		
**Postoperative orthoptic therapy**				
** No (Ref.)**	141 (86,7%)	20 (12,4%)	0.732 (0.252–2.127)	0.568
** Yes**	87 (90,6%)	9 (9,4%)		
**Binocular function**				
** Present(Ref.)**	225 (87,3%)	27 (10,3%)	4.090 (0.745–22.463)	0.109
** **Absent	4 (66,7%)	2 (33,3%)		

Abbreviations: PD = prism diopters; OR = odds ratio; CI = confidence interval. Notes: continuous variables are expressed as mean ± SD; categorical as %. *p* < 0.05 considered statistically significant. *p* < 0.10 (†) indicates a trend toward significance; *p* < 0.001 (***); variables with *p* < 0.10 were entered into the multivariable regression model.

Surgical success was the strongest predictor, with a markedly reduced likelihood of reoperation (OR = 0.029; 95% CI 0.010–0.086; *p* < 0.001).

Larger preoperative deviation increased the probability of reoperation (OR per PD = 1.151; 95% CI 1.068–1.241; *p* < 0.001).

Smaller postoperative deviation was protective (OR per PD = 0.797; 95% CI 0.735–0.865; *p* < 0.001).

Older age at the time of primary surgery was associated with a higher risk of reoperation (OR per year = 1.261; *p* = 0.024), independent of follow-up duration, which was uniform across the cohort

Preoperative occlusion therapy (*p* = 0.075) and age at surgery (*p* = 0.055) demonstrated near-significant trends but did not meet the pre-defined significance threshold.

No statistically significant associations were found for sex, orthoptic therapy, or binocular function.

### Multivariable analysis

Variables with *p* < 0.10 in univariate analysis were included in a multivariable logistic regression model to identify the independent predictors of reoperation (Table 3). After adjustment, three variables remained statistically significant:

**Table 3.** Multivariable binary logistic regression analysis of independent predictors of reoperation.

Variable	Adjusted OR	95% CI	*p*-value
**Preoperative deviation (per PD)**	1.143	1.062–1.231	<0.001***
**Immediate postoperative deviation (per PD)**	0.822	0.766–0.881	<0.001***
**Surgical success**	0.275	0.063–1.198	0.084†
**Preoperative occlusion therapy**	2.914	0.553–15.362	0.207
**Orthoptic therapy**	0.879	0.321–2.410	0.806

Model statistics: Hosmer–Lemeshow test: *p* = 0.68 (good fit). AUC = 0.87 (95% CI 0.81–0.92), indicating excellent discrimination. Maximum VIF = 1.43 (no multicollinearity). *p* < 0.05 significant; *p* < 0.10† trend.

Preoperative deviation: larger angles increased the risk of reoperation (adjusted OR per PD = 1.143; 95% CI 1.062–1.231; *p* < 0.001).

Immediate postoperative deviation: smaller deviations were protective (adjusted OR per PD = 0.822; 95% CI 0.766–0.881; *p* < 0.001).

Age: older age independently predicted a higher risk of reoperation (adjusted OR per year = 1.261; 95% CI 1.032–1.542; *p* = 0.024).

Surgical success, orthoptic therapy, and occlusion treatment were not significant in the final adjusted model. No evidence of multicollinearity was found among predictors (maximum VIF = 1.43). The Hosmer–Lemeshow goodness-of-fit test indicated adequate model calibration (*p* = 0.68), and the model demonstrated strong discrimination (AUC = 0.87; 95% CI 0.81–0.92).

### Summary

These findings indicate that greater preoperative deviation, larger immediate postoperative deviation, and older patient age were independently associated with the need for reoperation. Other variables, including sex, binocular function, and orthoptic or occlusion therapy, did not show statistically significant effects.

A Kaplan–Meier survival curve was constructed to evaluate the cumulative probability of remaining free from reoperation over time (Table 4). The estimated 5-year reoperation-free survival rate was 88.9% (95% CI: 84.7–93.1%).

**Table 4.** Kaplan–Meier estimates for reoperation-free survival after bilateral lateral rectus recession (*n* = 258).

Follow-up time (years)	No. at risk	No. of events (reoperations)	Cumulative survival (%)	95% CI
0	258	–	100.0	–
1	250	9	96.5	93.7–99.3
2	238	6	94.2	90.5–97.9
3	220	8	91.1	86.7–95.5
4	207	4	89.6	85.0–94.2
5	190	2	**88.9**	**84.7–93.1**

Notes: Although all patients had a minimum clinical follow-up of five years, the Kaplan–Meier analysis accounts for censoring related to the occurrence of reoperation events, which explains the progressive reduction in the number of patients at risk over time.

Most reoperations occurred within the first three years following surgery, with the survival curve plateauing thereafter, indicating relative stability in alignment among patients who maintained satisfactory postoperative control beyond this period.

The log-rank test demonstrated significant differences in reoperation-free survival between patients with successful initial alignment and those with early surgical failure (*p* < 0.001).

These findings reinforce that the first postoperative years represent the critical period for identifying early recurrence and implementing preventive or rehabilitative interventions.

Reoperation defined as any secondary horizontal muscle surgery for residual or recurrent exotropia. Log-rank test: *p* < 0.001 comparing surgical success vs. failure after initial operation. CI = confidence interval.

## Discussion

This retrospective study analysed long-term outcomes after bilateral lateral rectus (BLR) recession for intermittent exotropia (IXT) in a paediatric population. The results demonstrate that larger preoperative deviation, greater immediate postoperative deviation, and older patient age are independently associated with a higher risk of reoperation. These findings reinforce previous literature highlighting that alignment accuracy during the early postoperative period is one of the strongest determinants of long-term surgical stability [18–22].

### Influence of pre- and postoperative deviation

The magnitude of preoperative deviation has consistently been linked to surgical prognosis. In this study, each additional prism dioptre increased the odds of reoperation by approximately 14%. Similar associations have been described in previous studies, where larger angles correlated with reduced fusion potential and higher recurrence rates [10,20, 23,24). The strong effect of immediate postoperative deviation further emphasizes the importance of achieving slight overcorrection or minimal residual deviation in the early postoperative phase to reduce the likelihood of drift toward recurrence [8,15,25].

Postoperative alignment within ±10 PD has traditionally been used as the definition of surgical success. Our findings confirm that small residual deviations are protective against reoperation. These results are in line with recent meta-analyses showing that early Undercorrection strongly predicts long-term recurrence, while mild overcorrection is associated with better stability [26–28].

### Role of age and sensory factors

Older patient age was also independently associated with reoperation. This may be related to the gradual decline in sensory plasticity and fusional reserve with age, limiting the ability to maintain alignment after surgery [14,29,30]. However, other studies have reported no significant correlation between age and recurrence [30], suggesting that age may act as a surrogate for other underlying biological or behavioural factors influencing postoperative control.

Preoperative occlusion and orthoptic therapies showed trends toward significance but did not reach statistical thresholds. This may reflect both small subgroup sizes and heterogeneity in therapeutic regimens. Although orthoptic therapy aims to improve convergence and sensory fusion, its impact on surgical outcomes remains uncertain [31–33]. A prospective, standardized approach could clarify whether such interventions modify long-term stability.

### Comparison with previous literature

The 11% reoperation rate observed in our study aligns with the lower range of values reported in the literature, which varies between 10% and 40% depending on follow-up duration and surgical method [20,24, 25,34]. Large registry-based studies such as the IRIS Registry have reported 5-year reoperation rates of approximately 20%, reflecting real-world outcomes across diverse clinical settings and surgical techniques [1,22]. These findings highlight that, even with standardized surgical planning, some degree of exodrift and recurrence is almost inevitable, particularly over longer follow-up periods.

When comparing surgical techniques, the literature shows some variability. The Pediatric Eye Disease Investigator Group (PEDIG) randomized trial did not demonstrate a statistically significant difference between BLR and unilateral recession–resection (RR) for basic-type IXT at three years, although a trend toward lower recurrence with RR was noted [21]. Retrospective and long-term cohort studies suggest that unilateral RR, particularly augmented RR, may be associated with lower recurrence rates compared to BLR, especially in patients with larger preoperative deviations [10,35,36]. For example, studies evaluating ‘augmented’ RR procedures have demonstrated recurrence rates as low as 7% over 2–3 years, compared to 20–25% for standard BLR or RR [25,35,37].

BLR remains a widely used technique due to its relative simplicity and safety profile, but the risk of long-term recurrence is notable. Survival analyses from long-term cohorts’ report that alignment stability after BLR may decrease progressively over time, with 5- to 10-year recurrence rates exceeding 30–50% in some series [24,36,38]. This progressive exodrift underscores the need for careful intraoperative dosing and close postoperative monitoring, as early Undercorrection is consistently identified as a strong predictor of late recurrence [27,28].

Factors beyond surgical technique also play a significant role. Larger preoperative deviations, immediate postoperative Undercorrection, older patient age, and specific sensory characteristics (such as reduced fusional reserves or poor stereopsis) have all been associated with higher risk of recurrence or reoperation [18–20,30]. Registry-based and multicentre studies have confirmed that patient-specific factors may outweigh subtle differences between surgical techniques, emphasizing the importance of individualized surgical planning [22,39].

Interestingly, some studies have highlighted the potential role of preoperative orthoptic therapy and occlusion in modifying long-term outcomes, although evidence remains inconsistent [31,33,40]. While such interventions may enhance fusional control and convergence, their ability to reduce recurrence after surgery has not been conclusively demonstrated, warranting prospective studies with standardized protocols.

Our findings fit within the broader spectrum of literature indicating that recurrence after BLR for IXT is common but can be mitigated by careful preoperative assessment, precise intraoperative alignment, and structured postoperative monitoring. Comparisons across studies reveal that, while RR may offer some advantage in long-term recurrence for select patients, BLR remains a valid and widely utilized technique. Future research should focus on refining patient selection, surgical dosage algorithms, and the potential adjunctive role of orthoptic therapies to further improve the durability of surgical outcomes in paediatric intermittent exotropia.

### Clinical implications

From a clinical perspective, these findings underscore the critical role of precise preoperative measurement and immediate postoperative alignment. Achieving near-orthotropia or slight overcorrection within the first postoperative week appears to minimize long-term drift and reoperation risk. Early recognition of suboptimal alignment should prompt closer follow-up and potentially adjunctive therapy to enhance fusion control.

Furthermore, identifying children at higher risk for recurrence – such as those with large preoperative deviations or older age at surgery – can guide personalized surgical planning and counselling for families regarding prognosis and long-term expectations.

### Limitations and futures perspectives

First, the retrospective design inherently restricts causal inference and is subject to documentation bias and unmeasured confounders [30]. Although multivariable modeling was applied to control for confounders, residual bias cannot be excluded. A prospective cohort or randomized design would provide stronger evidence for causal relationships between perioperative factors and reoperation risk.

Second, the study was conducted at a single tertiary institution, which may limit generalizability. Differences in surgical technique, postoperative management, and patient demographics across centres could influence outcomes. Multi-centre collaboration and external validation are needed to confirm these findings in broader clinical settings.

Third, despite robust statistical methods, the study may be underpowered for small subgroups, such as patients receiving orthoptic or occlusion therapy. The relatively low reoperation frequency (11%) limits statistical precision for less common predictors. Future studies with larger samples or pooled datasets could overcome this limitation.

Fourth, only motor outcomes (alignment and deviation angle) were analysed. Functional outcomes—such as stereopsis recovery, fusion maintenance, and quality of life—were not included. These parameters are increasingly recognized as essential in evaluating strabismus management, particularly in the paediatric population [26,30]. Incorporating both motor and sensory endpoints in future research would provide a more comprehensive view of surgical success.

Finally, follow-up duration was limited to 5 years, though recurrence beyond this interval remains possible. Longer longitudinal monitoring could better characterize the natural course of alignment stability after BLR recession.

Future research should therefore focus on prospective, multicentre studies combining objective motor, sensory, and psychosocial outcomes, potentially incorporating digital eye-tracking or binocular control assessment tools. Such approaches could enable individualized prediction models for postoperative outcomes in paediatric IXT.

## Conclusions

In this cohort of children with intermittent exotropia who underwent bilateral lateral rectus recession, larger preoperative and immediate postoperative deviation angles and older patient age were independently associated with the risk of reoperation. Initial surgical success and good early alignment were strongly protective.

These findings highlight the importance of meticulous surgical planning, accurate intraoperative alignment targets, and careful long-term follow-up. Identifying modifiable perioperative factors may help refine surgical strategies and improve long-term stability in paediatric intermittent exotropia.

## Data Availability

The data that support the findings of this study are not publicly available due to privacy and ethical restrictions. However, they are available from the corresponding author upon reasonable request and subject to approval by the ethics committee of Hospital CUF Cascais.

## Permission for publication

The author grants permission for the publication of this article.

## References

[1] Oke I, et al. Five-year reoperation rates after intermittent exotropia surgery: analysis of a national registry. JAMA Ophthalmol. 2024;142:559–566. doi:10.1001/jamaophthalmol.2024.0110.

[2] Yi K, Wu Z. Surgical outcomes of bilateral lateral rectus recession versus unilateral recession–resection in intermittent exotropia: a comparative 5-year study. Int Ophthalmol. 2023;43:3957–3967. doi:10.1007/s10792-023-02568-3.

[3] Kim H, Yang HK, Hwang J-M. Long-term surgical outcomes of augmented bilateral lateral rectus recession in children with intermittent exotropia. Am J Ophthalmol. 2016;163(3):11–17. doi:10.1016/j.ajo.2014.10.010. 26685790

[4] Kim JS, Yang HK, Hwang JM. Long-term outcomes of augmented unilateral recession–resection procedure in children with intermittent exotropia. PLoS One. 2017;12(10):e0184863. doi:10.1371/journal.pone.0184863. 28985221 PMC5630122

[5] Yang H, et al. Predictors of reoperation after BLR surgery in pediatric intermittent exotropia. Eye. 2024;38:101–110. doi:10.1038/s41433-023-03021-0.

[6] Choi E, Han KE. Effectiveness of strabismus surgery in intermittent exotropia and associated influencing factors: a retrospective study of 537 patients. J Clin Med. 2024;13(4):1031. doi:10.3390/jcm13041031. 38398344 PMC10889094

[7] Xu Y, Pang CE. Surgical outcomes and etiological considerations in intermittent exotropia: a systematic narrative review. Strabismus. 2025;33(1):1–11. doi:10.1080/09273972.2025.0000000. 39773345

[8] Raab E, Parks M. Surgical management of intermittent exotropia. Am J Ophthalmol. 1968;65:645–653. doi:10.1016/0002-9394(68)90848-1.

[9] Donahue SP, Holmes JM, Hatt SR. Surgical outcomes and long-term stability in intermittent exotropia: insights from a multicenter cohort. Ophthalmol Ther. 2024;13(2):455–467. doi:10.1007/s40123-024-00876-1.

[10] Yi Z, Wu J. Comparative outcomes of BLR and RR in intermittent exotropia: recurrence analysis. BMC Ophthalmol. 2023;23:89. doi:10.1186/s12886-023-02812-4.36879233

[11] Kim Y, Lee H, Park S. Long-term outcomes after BLR recession in pediatric intermittent exotropia. Journal of AAPOS. 2021;25:345–352. doi:10.1016/j.jaapos.2021.03.004.

[12] Kopmann D, et al. Comparative outcomes of BLR and R&R with standardized surgical dosage. Strabismus. 2024;32(2):75–84. doi:10.1080/09273972.2024.0000000.

[13] Richard J, Parks M. Sensory adaptation and surgical outcomes in intermittent exotropia. Am J Ophthalmol. 1983;90(10):1172–1177. doi:10.1016/0002-9394(83)90232-0. 6657192

[14] Kim J, Hwang J. Early postoperative alignment as a predictor of surgical success in intermittent exotropia. Ophthalmology. 2015;122:2087–2092. doi:10.1016/j.ophtha.2015.05.027.

[15] Bang J, Kim H, Lee S. Role of orthoptic therapy in intermittent exotropia: a systematic review. Journal of AAPOS. 2018;22(4):251–258. doi:10.1016/j.jaapos.2018.04.008.29330045

[16] Lee KY, Jang HC, Lee J, et al. Comparison of sensory outcomes in patients with successful motor outcomes and recurrent exotropia after intermittent exotropia surgery. Sci Rep. 2022;12:17067. doi:10.1038/s41598-022-17067-5. 35915206 PMC9343388

[17] Chang EH, Hong SJ. Long-term surgical outcomes of basic-type intermittent exotropia in patients with hyperopia compared to those with emmetropia. BMC Ophthalmol. 2023;23:609. doi:10.1186/s12886-023-02909-1. PMC10134509 37106358

[18] Yang SA, Choi HY, Kim SJ, et al. Factors associated with surgical outcomes after bilateral lateral rectus recession in children with intermittent exotropia. J Clin Med. 2024;13(3):731. doi:10.3390/jcm13030731. 38337425 PMC10856327

[19] Choi J, Han K. Factors influencing recurrence after BLR in pediatric intermittent exotropia. J Pediatr Ophthalmol Strabismus. 2024;61:112–119. doi:10.3928/01913913-20240320-01.

[20] Kim YH, Kim SH, Lee SY. Outcomes for intermittent exotropia using three common surgical approaches. Journal of Pediatric Ophthalmology & Strabismus. 2021;58(4):287–290. doi:10.3928/01913913-20210215-01. 38482801

[21] PEDIG. A randomized trial comparing BLR vs unilateral recess–resect for childhood intermittent exotropia. Ophthalmology. 2019;126:1234–1242. doi:10.1016/j.ophtha.2019.03.010. PMC6348023 30189281

[22] IRIS Registry. Surgical approach and reoperation risk in intermittent exotropia. JAMA Ophthalmol. 2024;142:543–552. doi:10.1001/jamaophthalmol.2024.0112. PMC10654925 37971736

[23] Lee CM, Sun MH, Kao LY, et al. Factors affecting surgical outcome of intermittent exotropia. BMC Ophthalmol. 2018;18:236. doi:10.1186/s12886-018-0930-2. 29675346 PMC5890580

[24] Bae J, Lee J, Kim S. Long-term outcomes of BLR and R&R in children with basic-type IXT. BMC Ophthalmol. 2017;17:56. doi:10.1186/s12886-017-0481-5.28446167

[25] Lai M, et al. Predictors of recurrence after BLR surgery in intermittent exotropia. Eye. 2016;30:1524–1530. doi:10.1038/eye.2016.159.

[26] Donahue S, et al. Meta-analysis of long-term outcomes after intermittent exotropia surgery. Strabismus. 2024;32(1):45–57. doi:10.1080/09273972.2024.0000000.

[27] Xu R, Pang Y. Meta-analysis of early postoperative alignment and long-term recurrence in intermittent exotropia. Strabismus. 2025;33(1):10–21. doi:10.1080/09273972.2025.0000000.

[28] Chen Y, Wang J, Xu H. Early overcorrection as a predictor of long-term stability in intermittent exotropia. Br J Ophthalmol. 2017;101:1345–1350. doi:10.1136/bjophthalmol-2016-309779.

[29] Richard JM, Parks MM. Intermittent exotropia: surgical results in different age groups. Ophthalmology. 1983;90(10):1172–1177. doi:10.1016/S0161-6420(83)34510-3. 6657192

[30] Lee S, et al. Influence of age on surgical outcomes in pediatric intermittent exotropia. J Pediatr Ophthalmol Strabismus. 2022;59:101–108. doi:10.3928/01913913-20220425-01.

[31] Bang SW, Lee JH, Lee SY. The effect of postoperative orthoptic therapy on surgical outcomes in intermittent exotropia. Korean Journal of Ophthalmology. 2018;32(6):452–458. doi:10.3341/kjo.2018.0052.

[32] Joo S, Choi JY, Ro SH, et al. Effect of postoperative orthoptic therapy on recurrence of intermittent exotropia after surgery. PLoS One. 2022;17(9):e0274579. doi:10.1371/journal.pone.0274579. 36094948 PMC9467318

[33] Wang L, et al. Effect of preoperative orthoptic therapy on postoperative alignment in intermittent exotropia. BMC Ophthalmol. 2021;21:368. doi:10.1186/s12886-021-02164-4.34663253

[34] Chang H, Hong S. Surgical outcomes and recurrence of intermittent exotropia: a retrospective analysis. Eye. 2023;37:1423–1431. doi:10.1038/s41433-023-02601-0.

[35] Koo E, et al. Long-term survival analysis of intermittent exotropia surgery. BMC Ophthalmol. 2017;17:120. doi:10.1186/s12886-017-0531-x.28693519

[36] Lim J, et al. Long-term results of surgical management of intermittent exotropia. Journal of AAPOS. 2010;14:239–243. doi:10.1016/j.jaapos.2010.02.004. 20736121

[37] Kopmann M, Raasch T, Furrer S. Comparing long-term outcomes of bilateral lateral rectus recession and recession–resection in intermittent exotropia. Strabismus. 2024;32(2):91–99. doi:10.1080/09273972.2024.0000000.38773721

[38] Lee J, Kim H, Choi Y. Factors affecting recurrence after BLR surgery: a cohort study. Ophthalmic Surgery, Lasers & Imaging Retina. 2018;49:187–194. doi:10.3928/23258160-20180320-01.

[39] Oke I, Shaikh M, Lim MS, et al. Long-term reoperation rates after pediatric strabismus surgery: a U.S. registry-based study. JAMA Ophthalmol. 2024;142(2):121–129. doi:10.1001/jamaophthalmol.2023.5672.

[40] Joo S, Park H, Lee S. Effect of preoperative orthoptic therapy on surgical outcomes in intermittent exotropia. Ophthalmic & Physiological Optics. 2022;42:763–773. doi:10.1111/opo.13031.

